# Automatic Fracture Characterization Using Tactile and Proximity Optical Sensing

**DOI:** 10.3389/frobt.2020.513004

**Published:** 2020-12-02

**Authors:** Francesca Palermo, Jelizaveta Konstantinova, Kaspar Althoefer, Stefan Poslad, Ildar Farkhatdinov

**Affiliations:** ^1^School of Electronic Engineering and Computer Science, Queen Mary University of London, London, United Kingdom; ^2^Robotics Research, Ocado Technology, London, United Kingdom; ^3^The Alan Turing Institute, Programme – Artificial Intelligence, London, United Kingdom; ^4^Department of Bioengineering, Imperial College of Science, Technology and Medicine, London, United Kingdom

**Keywords:** sensing, haptic exploration, crack recognition, extreme environment, optical sensing, fiber-optics

## Abstract

This paper demonstrates how tactile and proximity sensing can be used to perform automatic mechanical fractures detection (surface cracks). For this purpose, a custom-designed integrated tactile and proximity sensor has been implemented. With the help of fiber optics, the sensor measures the deformation of its body, when interacting with the physical environment, and the distance to the environment's objects. This sensor slides across different surfaces and records data which are then analyzed to detect and classify fractures and other mechanical features. The proposed method implements machine learning techniques (handcrafted features, and state of the art classification algorithms). An average crack detection accuracy of ~94% and width classification accuracy of ~80% is achieved. Kruskal-Wallis results (*p* < 0.001) indicate statistically significant differences among results obtained when analysing only integrated deformation measurements, only proximity measurements and both deformation and proximity data. A real-time classification method has been implemented for online classification of explored surfaces. In contrast to previous techniques, which mainly rely on visual modality, the proposed approach based on optical fibers might be more suitable for operation in extreme environments (such as nuclear facilities) where radiation may damage electronic components of commonly employed sensing devices, such as standard force sensors based on strain gauges and video cameras.

## 1. Introduction

An important task often performed in remote hazardous environments is the detection of mechanical fractures on the objects, such as containers, tanks, pipes, and other technical systems used for keeping chemical and radioactive waste. A crack may be caused by physical damage or material degradation over time or environment changes (e.g., temperature or pressure). The effects of non-detected fractures may lead to larger macro-scale catastrophic failures making the cracked surface mechanically weak to perform its function.

Conventional automatic crack detection methods applied in industry to inspect large mechanical structures rely on acoustic methods (Chakraborty et al., 2019), use X-ray scanning (Barhli et al., 2017; Naragani et al., 2017), apply eddy currents techniques (Yao et al., 2014), or explore changes in a system's motion dynamics (Lu and Chu, 2011; Nicoletti et al., 2018). Such techniques require specialized and costly equipment and well-trained technical staff making their usage in extreme environments (i.e., decommissioning of radioactive waste) less beneficial or even impossible.

Rapid development of computer vision and machine learning led to the introduction of multiple vision-based tools for mechanical fracture detection that we briefly review below. Chen and Jahanshahi (2017) proposes a fusion between a convolutional neural network and a Naive Bayes to analyse video frames for crack detection in nuclear reactors. The framework achieves a 98.3% hit rate against 0.1 false positives per frame. Schmugge et al. (2016) suggested a crack detection method for nuclear power plant inspection videos by fine-tuning a deep neural network for detecting local patches containing cracks which are then grouped in spatial-temporal space for group-level classification which obtains an increase of 40% in the F1-Score with respect to the compared methods. Iliopoulos et al. (2015) analyzed the evolution of a cracked concrete structure obtained by applying Digital Image Correlation, Acoustic Emission, and Ultrasonic Pulse Velocity techniques. The results highlight the time of onset and location that the crack started to form as well as the width and depth of the cracks.

Vision based methods demonstrate high detection accuracy and they are easy to implement in telerobotics applications as cameras are essential parts of the remote inspection robots. However, vision-based methods can fail in remote environments with limited luminosity and video-cameras cannot operate in presence of strong radiation. Furthermore, vision-based methods are not capable of acquiring material properties, such as texture and hardness.

Our work proposes to use tactile and proximity sensing for mechanical cracks detection. In contrast to the visual modality, tactile, and proximity sensing can provide important information on material properties, such as shape, texture, and hardness (Huet et al., 2017; Yuan et al., 2017; Kaboli and Cheng, 2018). Tactile sensors were efficiently used to characterize different materials in robotic teleoperation. Liu et al. (2012, 2015) implemented a 6-axis force/torque finger-shaped sensor capable of estimating the instantaneous friction force and normal force to recognize physical properties of the surface of unknown objects. Average classification accuracy of 88.5% is obtained when implementing a naïve Bayes classifier on 12 different texture surfaces. Feng et al. (2018) proposed a new method, called Active Prior Tactile Knowledge Transfer (APTKT) to re-implement tactile knowledge of previously explored objects which improves the discrimination accuracy by over 20%. A multi-modal tactile sensor (BioTac, developed by SynTouch^1^) was used by Wong et al. (2014) to estimate the order of curvature and footprint dimensions explored with various movements (distal-proximal stroke, radial-ulnar stroke, etc.) of the robotic finger. Fishel and Loeb (2012) proposed a Bayesian exploration which selects the optimal movements based on previous experience to recognize 117 different textures. Kaboli et al. (2016) propose an online tactile transfer learning method to re-use previously learned tactile models to discriminate new textures with limited numbers of training samples. An expanded tactile sensors module was implemented for recognizing the alphanumeric characters inscribed on rubber stamps in Lee et al. (2006). The stiffness of objects was investigated by Konstantinova et al. (2017) implementing a hybrid force and proximity finger-shaped sensor achieving 87% classification accuracy on a set of household objects with different stiffness values. Drimus et al. (2014) proposed a method to classify objects into rigid and deformable using dynamic time warping to compare the distance between time series of signals. An optical sensor was implemented by Huang et al. (2018) to detect target objects in dynamic environments prior to contact allowing the teleoperator to feel the object without an actual contact improving the benefits of touch interaction to the operator, without negative consequences of the robot contacting unknown geometric structures. Tomo et al. (2017, 2018) introduced uSkin, a soft-skin based sensor, which measures the applied force based on changes in the magnetic field for object shape recognition. Not many approaches use tactile sensing for crack detection and characterization. For additional research on tactile sensing and texture recognition, please refer to Kappassov et al. (2015) and Luo et al. (2017).

In this work, we propose a novel tactile sensing-based technique for mechanical fractures detection with the potential application to nuclear-decommissioning tasks performed by remotely operated robots. The nuclear power industry has been among the slowest to adopt advanced technologies (Wood, 2004; Bogue, 2011). Any instrumentation to be used in the nuclear environment must show robustness under the influence of nuclear radiation, match safety requirements and satisfy the highest industrial standards. The effects of radiation greatly vary and depend on several parameters, including the type of radiation and the total dose (Bogue, 2013). Our approach relies on optical fibers for data transmission from the sensor's measurement elements to the remotely located electronic unit. Optical fibers are among the devices that are less influenced in a nuclear environment since gamma radiation does not interfere with their basic sensing mechanism (Berghmans et al., 1999; Inaudi et al., 2001; Phéron et al., 2012). Berghmans and Decreton (1998) compared the gamma radiation response of three types of optical fiber temperature sensors. For the three sensor types, the transducer mechanism does not seem to be affected by gamma radiation. Fiber optic cables are expected to see greater use in the nuclear power industry, replacing electrical cables (Berthold, 1994; Hashemian, 2009). Several applications implementing fiber optic cables are already been realized. Kim et al. (2017) developed a fiber-optic based monitoring system for water temperature, water level and radiation level of spent nuclear fuel pool (SNFP) at a nuclear power plant. The performance test results show that individual sensors can measure the changes in real-time. Ball et al. (2012) described several measurement technologies with potential application to gas reactors. Among these, an optical-based pressure sensor based on the trajectory of the light in glass is analyzed. The polarization of light crossing the glass is created through stress-induced in the glass as a result of pressure. Through the fiber optic sensor, the pressure measurement can be found through the polarized light intensity.

Present work demonstrates how tactile and proximity sensing can be efficiently used to perform automatic crack detection. The proposed method uses machine learning techniques to detect cracks and bumps based on the deformation and proximity signals which are recorded during physical interaction between a custom-designed robotic finger and the remote environment, Konstantinova et al. (2017). In case a crack is detected, the proposed automated technique classifies its width. Both offline and online classifications are performed. A fiber optic sensor has been implemented for data acquisition because of the reduced dimensions (~55 mm), weight (~200 g), low cost, the strong immunity to electromagnetic interference and the improved environmental resistance. This approach may be implemented also in extreme environments (e.g., in nuclear plants), since gamma radiation does not interfere with the basic sensing mechanism of fiber optic-based sensors (Berghmans et al., 1999). In addition, the nylon component of the implemented sensor can be used in irradiation conditions with limitations as Morita and Seguchi (1983) presented. To the best of the authors' knowledge, this is one of the first works on fracture recognition based on hybrid fiber optical force/proximity sensors. The Present work is based on our previous results (Palermo et al., 2020) demonstrating the feasibility of a tactile sensor for cracks detection. The novelty of this work is the implementation of more accurate mechanical fracture detection and classification methods, and a corresponding comparative study. Additionally, this paper provides a detailed description of the tactile data collection, processing and real-time classification implementation.

## 2. Experimental Methodology

### 2.1. Tactile and Proximity Sensor

**Figure 1:**
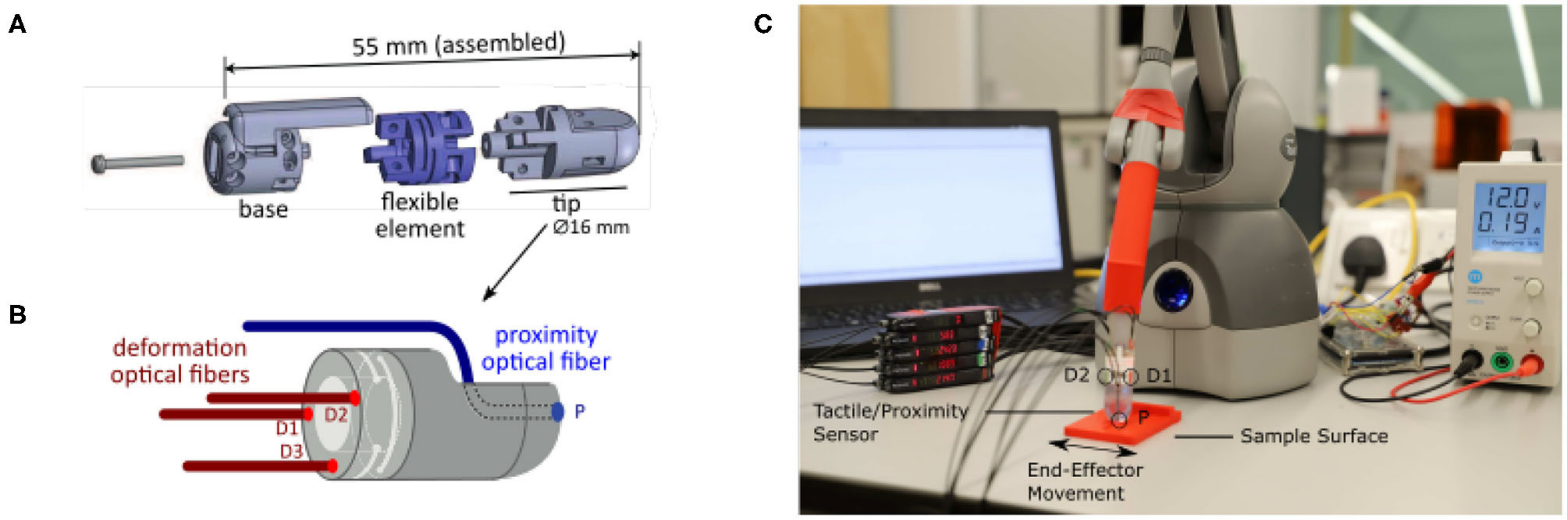
Hybrid Fiber Optical Force/Proximity fingertip sensor: **(A)** Visualization of the three different components of the sensor; **(B)** Close up visualization of the fiber optics operating principles. D1, D2, D3 indicates the three deformation optical fibers. P the proximity optical fiber. **(C)** The complete setup for the data acquisition. From left to right: Laptop, Keyence sensors, Touch Haptic device with 3d printed end effector, and Hybrid Fiber Optical Force/Proximity Sensor, Arduino board, and Power Supply.

In this work, the integrated force and proximity finger-shaped sensor described by Konstantinova et al. (2017) is used. The sensor is made of 3D printed rigid (VeroClear Glossy) and soft (Nylon—PA2200) components allowing it to bend during interaction with the environment, as shown in Figures 1A,B. All the components are printed with an SLS printer EOS P100. The sensor employs three pairs of optical fiber cables (D1, D2, D3) to measure the deformation of the flexible middle part based on the changes of the reflected light intensity. The fourth pair of optical fiber cables (P) is used to measure proximity, i.e., the distance between the tip of the finger sensor and nearby objects. The implemented proximity permits the shape recreation in 2D of the explored surface. The sensor is capable of measuring bending torque and normal contact force during physical interaction with the environment. As described in Konstantinova et al. (2017), the implemented sensor is able to detect three-axis force/torque signals and measure the distance to the explored object. The sensor measures normal force up to 4.5 N. The lateral torque values (around the x- and y-axes) reach a maximum of ±18 N/mm. The usage of nylon to print the flexible structure led to low hysteresis and high robustness. The proximity sensor (P) can measure distances up to 30 mm. The calibration method has been described in Konstantinova et al. (2016). Each pair of the sensor's fiber optic cables is attached to a Keyence FS-N11MN light-to-voltage transducer. Thus, the change of light intensity modulation is measured and, using a calibration matrix, converted to force, torque, and distance measurements.

### 2.2. Experimental Setup

To collect data and test the proposed crack detection algorithm, the tactile and proximity sensor, described in section 2.1, has been attached to the end-effector of a Touch desktop haptic interface (formerly known as Phantom Omni Geomagic) as shown in Figure 1C. The Phantom Omni was programmed to slide the tactile sensor along a static sample surface following a programmed periodic movement. Data from tactile and proximity sensors were recorded through an Arduino Mega ADK micro-controller, connected via a USB port, at 400 Hz. These data were later synchronized with the absolute position of the tip of the tactile sensor calculated through the encoder readings of the Phantom Omni. Data acquisition and control were implemented through dedicated software libraries (OpenHaptics and Robotic Operating System) running on an Ubuntu desktop computer. The material samples, as well as the Phantom Omni interface, were fixed to a laboratory desk to minimize any vibration and unwanted displacements.

### 2.3. Data Acquisition Protocol

**Figure 2:**
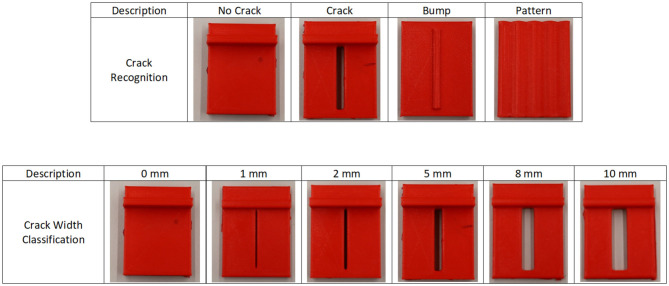
Visualization of the set of objects explored during the experiments. The set for the Crack Recognition Analysis is formed by no crack, crack, bump, and wavy pattern surfaces. The series for the Crack Width classification experiment is made up of the same fractured surface with distinct widths of 0, 1, 2, 5, 8, and 10 mm.

**Figure 3:**
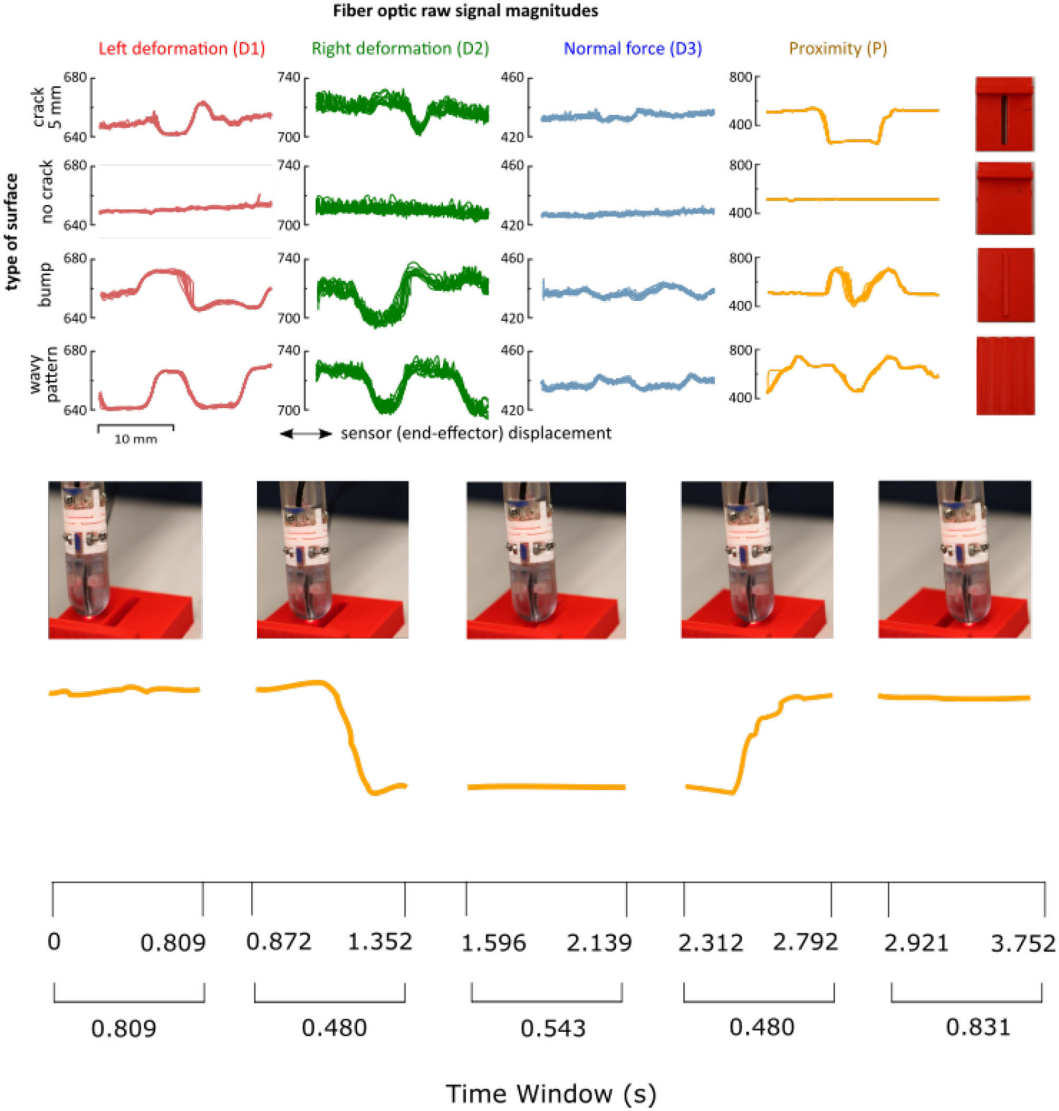
**(Top)** Raw measurements from the four sensing elements of the sensor (deformations D1, D2, D3, and proximity P) for the set of surface patterns: “no crack,” “crack,” “bump,” and “wavy pattern.” Each column shows a different optical fiber signal. In red, the results of the left deformation (D1). In green, the data of the right deformation (D2). In blue, the normal deformation (D3). In yellow, the proximity data (P). **(Bottom)** The movement of the sensor is shown together with the corresponding proximity data.

In this work, machine learning techniques are employed for crack detection and crack width classification. A set of 10 objects with different surfaces (no crack, cracks of different widths, a bump and a wavy pattern) were manufactured with PLA plastic using 3D printing technology (Ultimaker III, 0.2 mm layer height, 0.4 mm nozzle diameter). The wavy pattern consists of a repeated pattern of sine waves of 1mm amplitude and 5mm magnitude. The samples are shown in Figure 2. The types of these sample objects correspond to the classes implemented for training and testing the classifier. The Phantom Omni moved the tactile sensor across the sample objects: the periodic sliding has a magnitude of 1.6 cm and a frequency of 1,000 Hz. The average sliding velocity was 3.89 mm/s. The initial position of the tactile sensors was not controlled and varied from trial to trial and was set at ~5–10 mm from the crack edge. No normal force was applied by the sensor to the sampled surfaces except the force caused by the sensor weight (~200 g). Tactile and proximity signals were recorded for 12 repeated continuous sliding movements. This continuous recording was repeated five times. Figure 3 shows an example of raw data acquired on “no crack,” “crack,” “bump,” and “wavy pattern” for a continuous recording. For brevity, only the data acquired during sliding on different surfaces are shown.

### 2.4. Experimental Dataset

The data-set generated and used in this study is publicly available on the figshare repository “Automatic Fracture Database”^2^. The data is organized in a nine column format, corresponding to the following chronological measurements: sensor displacement, sensing elements signals (D1, D2, D3, P), the identification number of the current experiment, the number of the measurement trials (single sensors movement), the type of the surface explored (0 = no crack, 1 = crack, 2 = bump, and 3 = wavy pattern), and the direction of movement (0 = right, 1 = left).

## 3. Data Analysis for Crack Detection

The goal of the proposed algorithm is to detect and characterize mechanical fractures, such as cracks, based on the deformation and proximity data recorded from the sensors. The time history of the deformation and proximity data is recorded. Preprocessing step and features extraction are performed. The resultant output is used as an input for the classification algorithm.

### 3.1. Pre-processing

**Figure 4:**
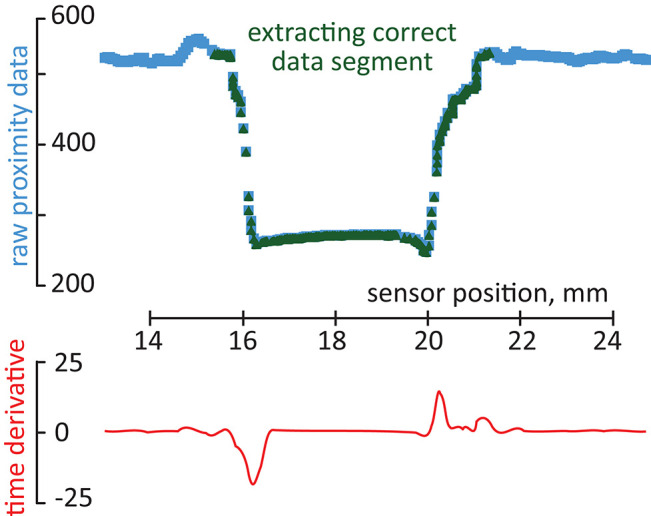
Example of correct segment extraction. The complete measurement trial is displayed in blue. In green, the data points corresponding to the changes in the sensor-sample interaction mechanics. In red discrete-time derivative for proximity measurement.

**Figure 5:**
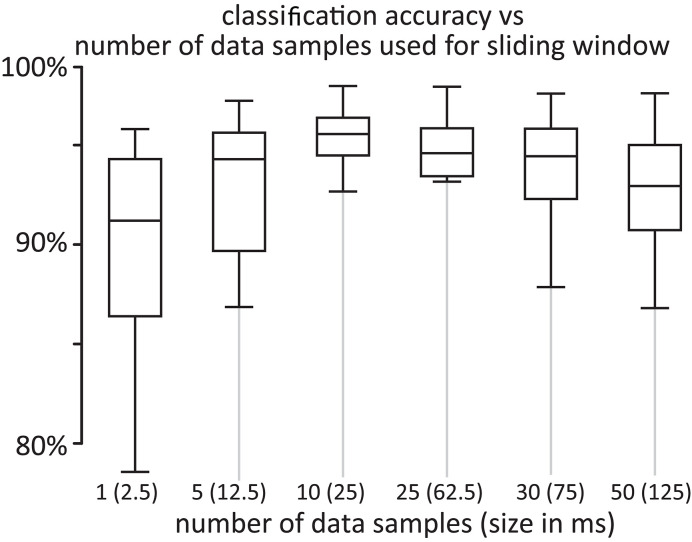
Comparison of classification accuracy of different moving window size to compute the derivatives for the automatic preprocessing step. Time window of *n* = 10 data samples achieves better classification accuracy.

The goal of preprocessing was to prepare datasets containing information for the mechanical features, such as crack, bump, and wavy pattern and exclude not relevant datapoints (i.e., recording of the sensors sliding on a flat surface before and after interacting with the bump or crack). The preprocessed labelled datasets were then used for training process and cross-validation test. The preprocessing was fully automatic and was performed on the data collected from the haptic manipulator (sensor displacement) and optical sensing elements integrated in the sensor (deformations D1, D2, D3 and proximity P). The sensor's position data (obtained from the Geomagic haptic device) and the sensing elements data (D1–D3, P) were synchronized and sampled at 400 Hz. Prior the prepossessing stage the measurements of each trial (single sensor movement along the explored surface) were arranged in the following matrix:

(1) $$\begin{array}{l} \underset{m\times 5}{\text{M}}=\left[{\text{x}}^{⊤}\;{\text{d}}_{1}^{⊤}\;{\text{d}}_{2}^{⊤}\;{\text{d}}_{3}^{⊤}\;{\text{p}}^{⊤}\right], \end{array}$$

with **x**, **d_1_**, **d_2_**, **d_3_**, and **p** vectors of size *m* × 1 representing single trial recordings (time history) of the sensor's displacement, three deformation signals and proximity signal, correspondingly, and *m* the number of data points in a specific trial. The proximity data (**p**) of each measurement trial was used to extract the data points corresponding directly to a specific mechanical feature (crack, bump, wavy pattern). This allowed to create a subset of data containing only the information specific to the mechanical feature, and to exclude the data points at the start and the end of the recording. This process was performed automatically, based on the analysis of the discrete-time derivative of proximity sensing for a given time window, and extracting the data for which the derivative exceeded a pre-defined threshold. The average discrete-time derivative for proximity measurement was computed as

(2) $$\begin{array}{l} \Delta p_{i}=\frac{{\text{p}}_{i+n}-{\text{p}}_{i}}{n},\\ \;\bar{\Delta \text{p}}=\frac{1}{m-n}\overset{m-n}{\underset{i=1}{\sum }}\Delta p_{i} \end{array}$$

with Δ*p*_*i*_ a local discrete derivative of *i*th proximity signal at measurement based on *n* data points, **p**_**i**_ representing *i*th element of proximity measurement vector, and $\bar{\Delta \text{p}}$ representing the averaged discrete-time derivative of the proximity measurements. Then, the data points of all measurement signals for which $\Delta p_{i}>|\bar{\Delta \text{p}}|$ are extracted from each trial as they represent the changes in the sensor-sample interaction mechanics. Additional 10% of the original measurement data is added before and after the extracted points to ensure that the data is complete and represents the explored mechanical feature well. Figure 4 shows an example of the data extraction for one sliding movement on a crack object. The complete measurement trial is displayed in blue. In green, the data points corresponding to the changes in the sensor-sample interaction mechanics. In red, these represent the discrete-time derivative for proximity measurement. To determine the appropriate moving window size for computing the derivatives we performed sample classification tests with different sliding window sizes. Figure 5 shows the results of this test which demonstrated that a time window of 25 ms (containing *n* = 10 data samples), is sufficient to achieve better good classification accuracy.

### 3.2. Feature Extraction

Feature extraction was performed on each successive 25 ms time window with an increment of 5 ms. The size of the time window was selected based on the sampling frequency. Feature extraction is executed on windows of 10 data points with a window shift of 2 data points. The window length was empirically chosen through a grid search analysis. Time-domain features, including Mean Absolute Value (MAV) and Root Mean Square (RMS), were computed. The advantage of time-domain features is that they are fast to calculate since they do not require any mathematical transformation, e.g., into the frequency domain. On the other hand, they are sensitive to noise. These feature demonstrated high performance in previous surface Electromyography (sEMG) works of (Hakonen et al., 2015; Palermo et al., 2017).

### 3.3. Classification Algorithm

**Figure 6:**
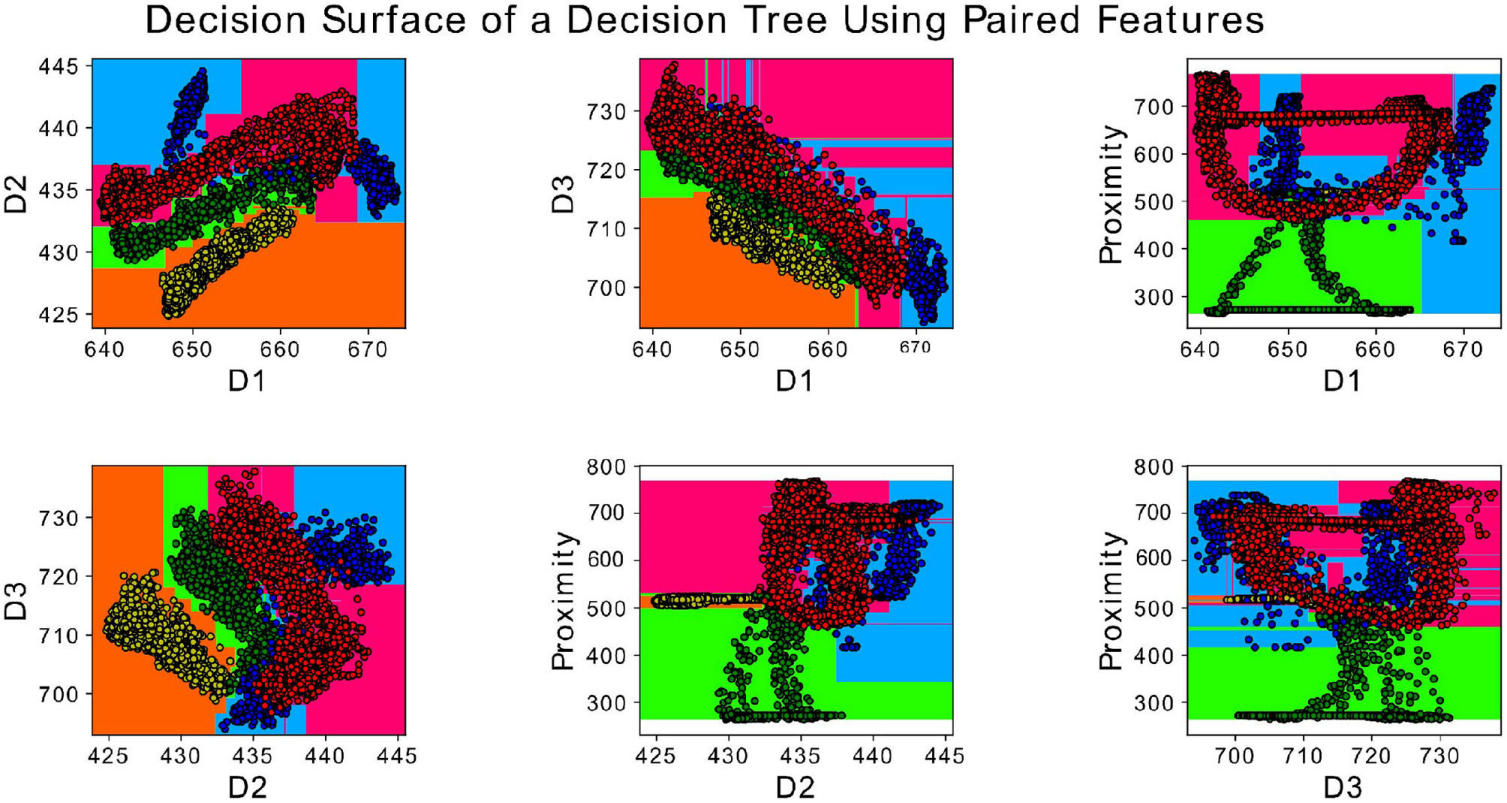
Decision surface of random forest classifier with four classes (no crack, crack, bump, and wavy pattern) for paired features: D1, D2, D3, and Proximity.

**Figure 7:**
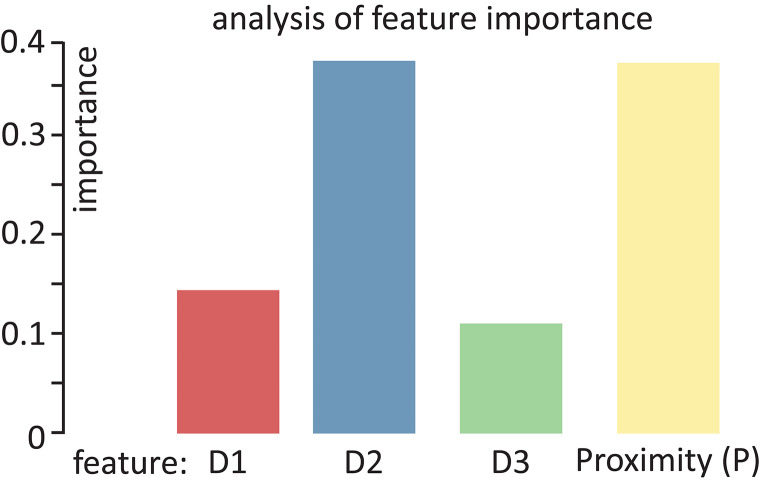
Feature importance analysis for the random forest classifier.

A set of classifiers was employed for the classification step: Random Forest with 100 trees, K-Nearest Neighbors (KNN) with five neighbors, and Quadratic Discriminant Analysis (QDA). Random Forest classifier (Breiman, 2001) can successfully handle high data dimensionality since it is both fast and insensitive to over-fitting. In addition, it was evaluated for remote sensing Belgiu and Drăguţ (2016). First, Random Forest with 1,000 trees was tested but it resulted in non-statistical relevant differences in respect to a Random Forest with 100 trees. It was then decided to discard it and use the Random Forest with 100 trees to increase the speed of the classification. The classification classes are equal to the type of surface explored (no crack, crack, bump and wavy pattern) for the surface crack recognition experiment and the width of the crack (0, 1, 2, 5, 8, and 10 mm) for the crack width classification. The complete data-set was then split 70% for training test and 30% for testing. Figure 6 shows an example of the decision surface of one of a decision tree of the Random Forest for paired features of Proximity (P) and Deformation data (D1, D2 and D3) with MAV feature. First, raw, MAV and RMS data were classified using only the proximity data (P) or the deformation signals (D1, D2, D3). During the experiments, it was found that implementing the four dimensionality features together (P, D1, D2, D3) over-fitted the classifier. The features importance analysis was performed to avoid over-fitting. Figure 7 shows the calculated feature importance. Among the four features. D3 is the least decisive one for the random forest classifier. Thus, the random forest was later trained and tested on proximity data (P) together with D1 and D2 deformation signals. Each observation was trained on itself and tested against the rest of the set one at a time (e.g., observation 2 was trained on itself and tested against observations 1, 3, 4, and 5) for intersession investigation. In total, 20 results for each analyzed feature were obtained. Kruskal-Wallis statistical analysis, which indicates if the data samples come from the same distribution, was performed on the whole set of results.

## 4. Results

**Figure 8:**
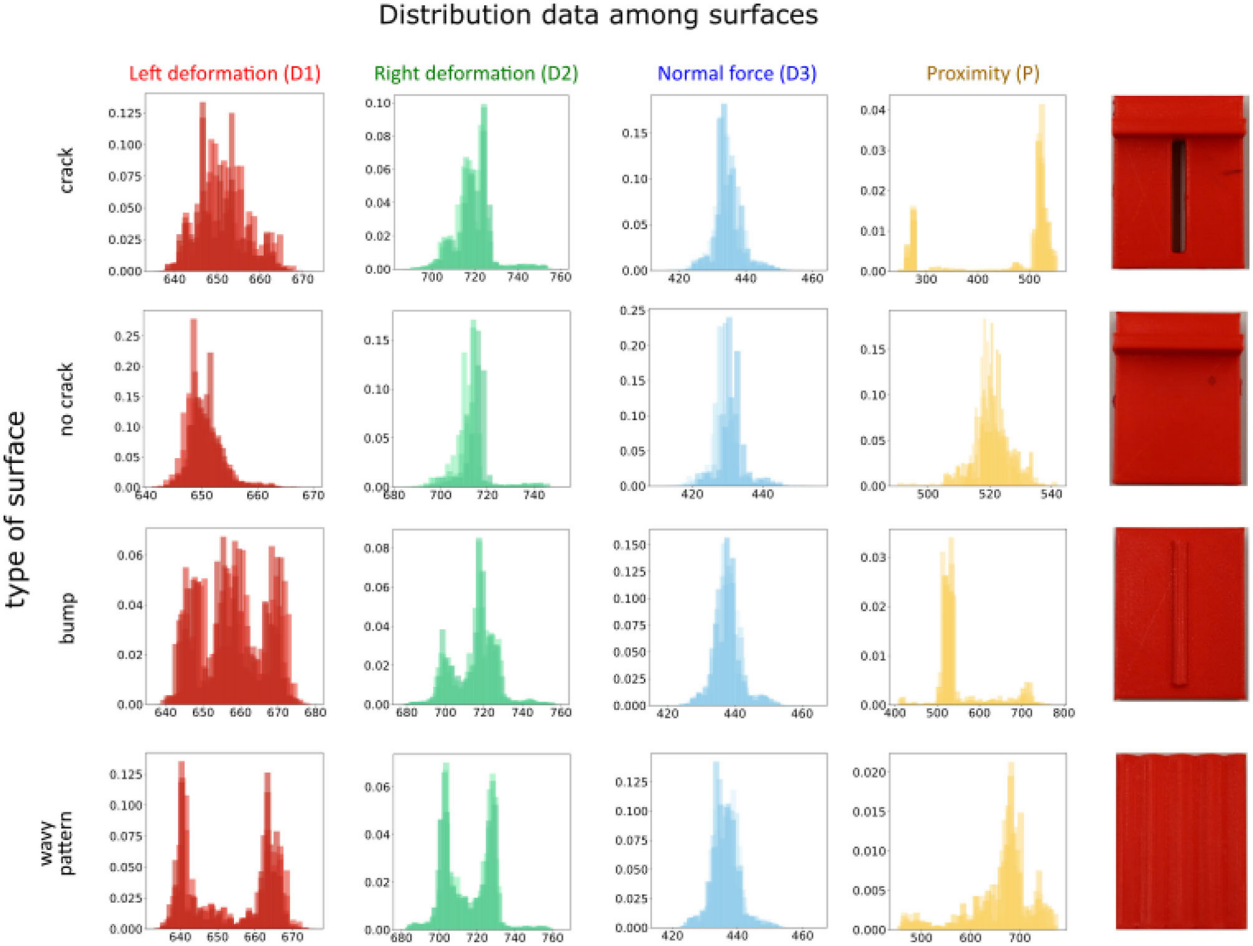
Measurements distribution for sensing elements D1, D2, D3, and P for “crack,” “no crack,” “bump,” and “wavy pattern” samples. Each plot shows distribution collected from six measurement trials.

Figure 8 shows the distribution of data on the different surfaces of 6 of the 12 repetitions, for brevity. The common response among the repetitions permits to have no dependence on the sensor starting position and movement.

### 4.1. Crack Recognition

**Figure 9:**
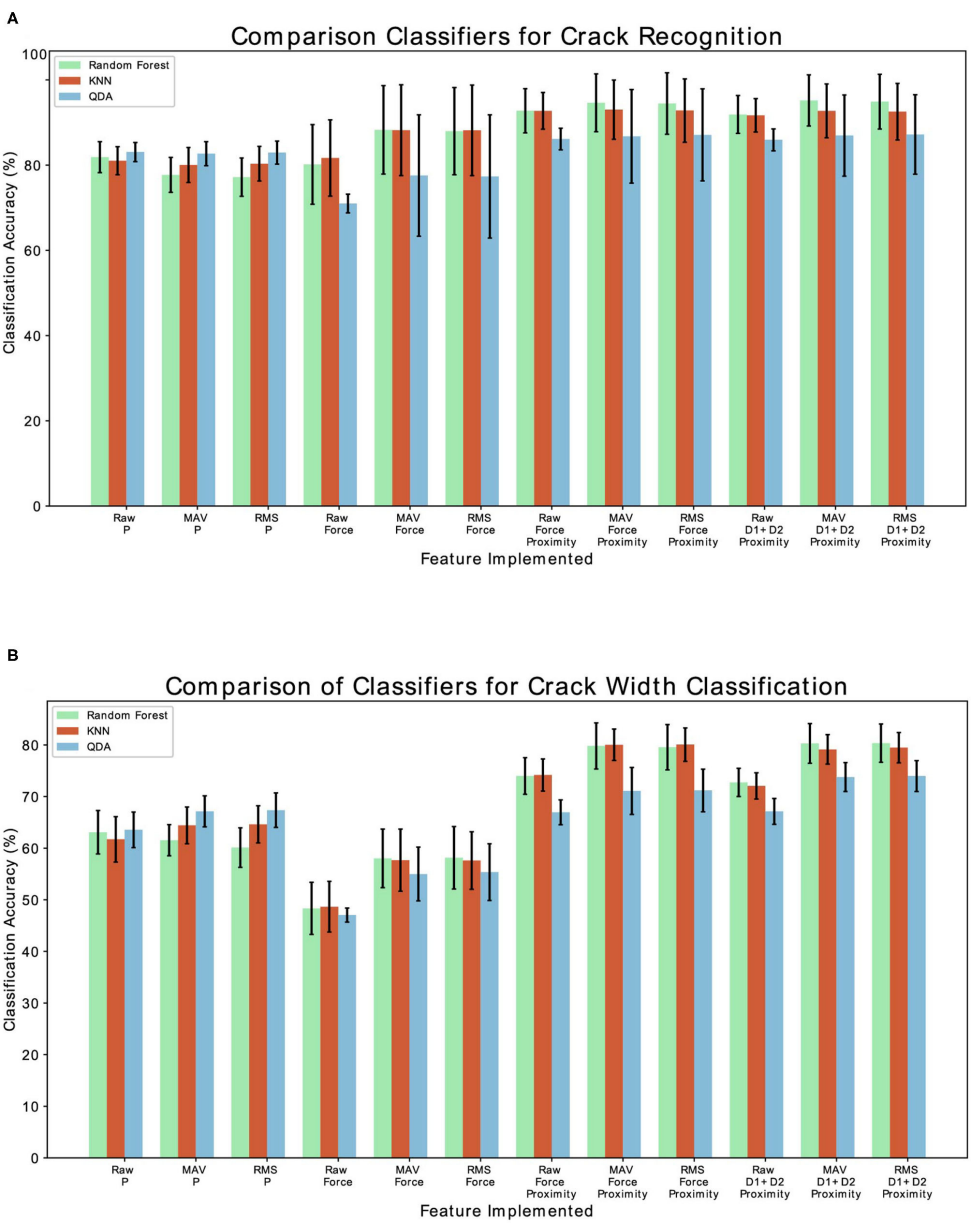
**(A)** Comparison of the three implemented classifiers (Random Forest, QDA, and KNN) for fracture recognition classification. **(B)** Comparison of the three implemented classifiers (Random Forest, QDA, KNN) for crack width classification.

**Figure 10:**
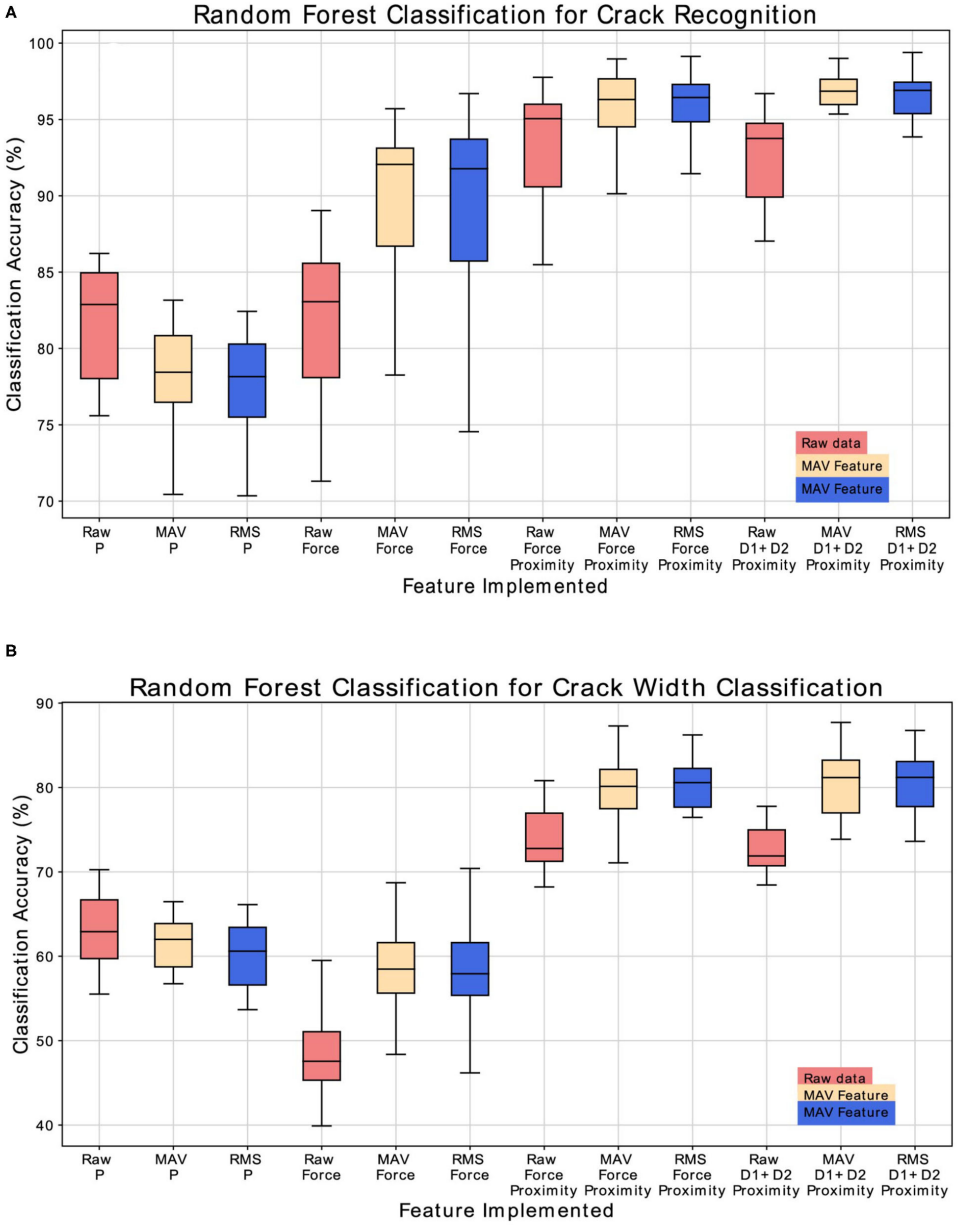
**(A)** Classification accuracy results for the crack recognition. **(B)** Classification accuracy results for the crack width classification analysis.

**Figure 11:**
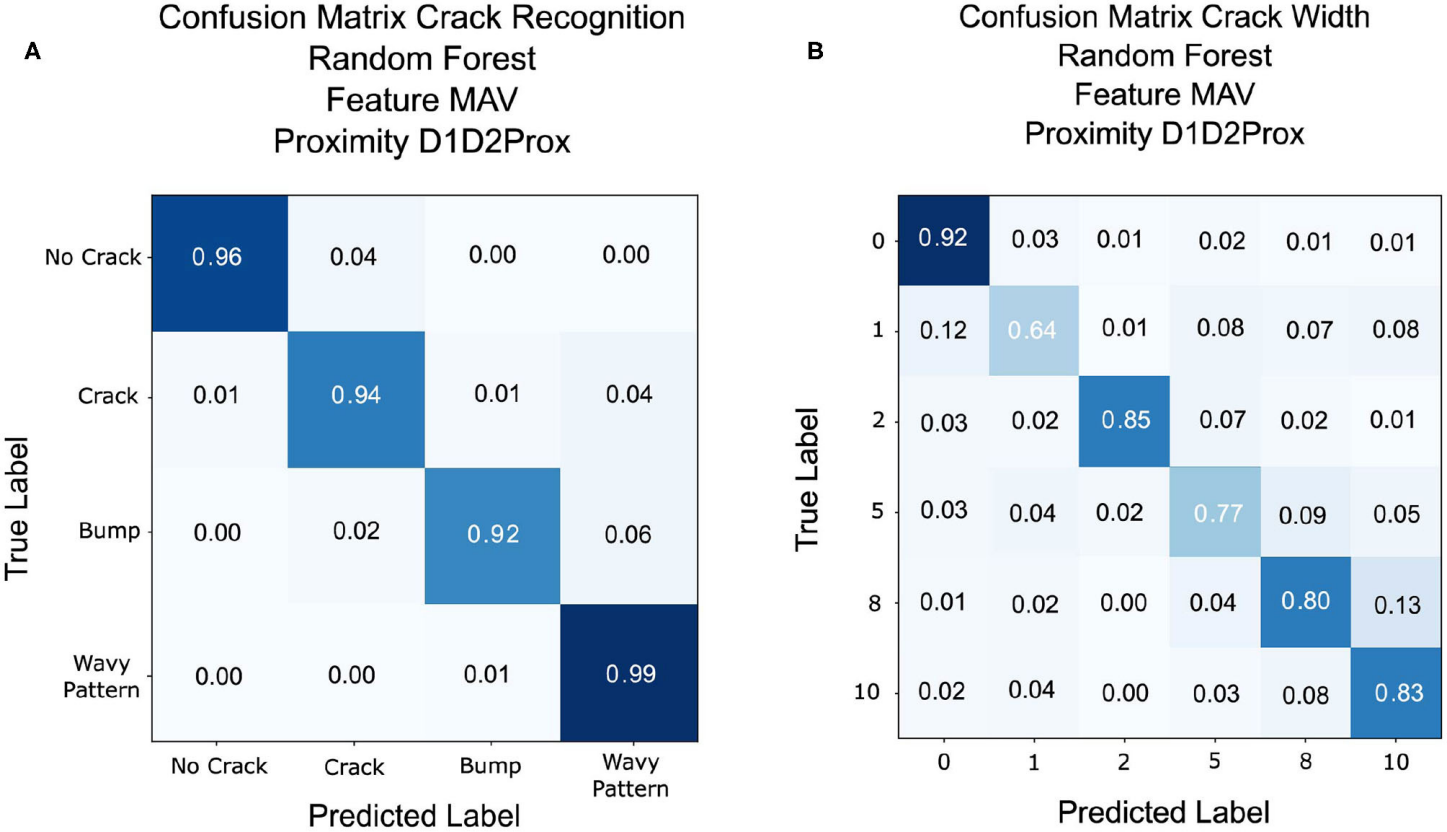
**(A)** Confusion matrix result for crack recognition with Random Forest classification with MAV feature and left and right displacements combined with proximity data. **(B)** Confusion matrix result for crack width classification with Random Forest classification with MAV feature and left and right displacements combined with proximity data.

The goal of the Crack Recognition experiment is to recognize the presence of a crack in the object. Figure 9A shows the results of the classification with the implemented classifiers. Random Forest achieves the best classification accuracy using the implemented feature of MAV and RMS and considering the left and right displacement of the sensor (D1, D2) together with the proximity data (P). The second best classifier is KNN, which is expected, since the various class (nocrack, crack, bump, wavy pattern) data are distributed in close proximity to each other, as shown in Figure 6. For brevity, only the results of the Random Forest classifier are shown in the following tables and figures. Figure 10A shows the complete results for the classification analysis. Table 1 shows that the lowest classification accuracy of 77% is obtained when classifying MAV or RMS data only considering the proximity data. Whereas, the best classification accuracy of 94% is achieved when implementing the MAV or RMS feature for the left and right displacement of the sensor and the proximity data. Using only deformation or only proximity data may be sufficient to train the classifier. However, better results are obtained when increasing the dimensionality of the classifier and considering proximity (P) together with the left and right displacement of the sensor (D1, D2). Thus, Implementing the whole deformation signals together with the proximity data brings little or nothing improvement to the classification accuracy in respect to using a feature with one less dimensionality. Figure 11A shows the results for the crack recognition. The most difficult surface to classify for the algorithm is the bump surface since it is comparable to the wavy pattern one. The Kruskal-Wallis test was performed on the results of the classification analysis of the different features and the value obtained (*p* < 0.001) indicates that the null hypothesis of having all data samples from the same distribution is rejected. Thus, there are significant differences between the implemented features.

**Table 1:** Complete classification accuracy for crack recognition experiment with Random Forest classifier.

**Implemented feature**	**Mean (%)**	**Standard deviation**	**Precision score (%)**	**Recall score (%)**
RAW—Proximity	81.86	3.64	82.66	81.86
MAV—Proximity	77.70	4.10	79.25	77.70
RMS—Proximity	77.17	4.48	79.07	77.18
RAW—Deformation (D1, D2, D3)	80.16	9.34	81.97	80.16
MAV—Deformation (D1, D2, D3)	88.27	10.37	90.71	88.27
RMS—Deformation (D1, D2, D3)	87.96	10.23	90.43	87.96
RAW—Deformation + Proximity	92.75	5.17	93.83	92.75
MAV—Deformation + Proximity	94.64	6.79	95.93	94.64
RMS—Deformation + Proximity	94.48	7.21	95.88	94.48
RAW—Deformation (D1, D2) + Proximity	91.88	4.44	92.76	91.88
MAV—Deformation (D1, D2) + Proximity	95.17	5.99	96.10	95.17
RMS—Deformation (D1, D2) + Proximity	94.90	6.42	95.93	94.90

### 4.2. Crack Width Classification

The scope of the crack width classification experiment is to classify the width in millimeters (mm) of the fracture of the explored object. Figure 9B shows the comparison of accuracy, recall score, and precision score of the implemented classifier. Random Forest classifier achieves the best classification accuracy, followed by KNN. As described in the previous section, class data are close in proximity to each other, which is why KNN obtains good results for this experiment. Figure 10B and Table 2 shows the complete results for the classification analysis. The lowest classification accuracy of 48.19% is obtained when classifying raw data with only deformation signals. Whereas, the best classification accuracy of ~80% is achieved when implementing MAV or RMS features with left and right deformation (D1 and D2) together with the proximity data. In this case, using only deformation or only proximity data is not sufficient to train the classifier. Figure 11B shows that the most difficult label to classify is the fracture of 1 mm width which can get mislabeled as a flat surface. This may be due to the fact that the fracture is so small that the left and right displacement are not big enough to trigger the recognition of the crack. Kruskal-Wallis results (*p* < 0.001) indicate statistically significant differences among results obtained when analyzing only deformation signals, only proximity data and both deformation and proximity data. In this case, instead of using a classifier, a regressor may be more appropriate to use since having a discrete class may not be the best solution when predicting the width of a fracture.

**Table 2:** Complete classification accuracy for crack width classification experiment with Random Forest classifier.

**Implemented feature**	**Mean (%)**	**Standard deviation**	**Precision score (%)**	**Recall score (%)**
RAW—Proximity	63.09	4.20	61.89	63.09
MAV—Proximity	61.54	3.02	62.00	61.54
RMS–Proximity	60.11	3.82	61.35	60.11
RAW—Deformation (D1, D2, D3)	48.33	5.05	45.63	48.33
MAV—Deformation (D1, D2, D3)	58.02	5.67	56.68	58.02
RMS—Deformation (D1, D2, D3)	58.13	6.05	56.71	58.14
RAW—Deformation + Proximity	73.97	3.55	74.47	73.97
MAV–Deformation + Proximity	79.81	4.46	81.27	79.81
RMS—Deformation + Proximity	79.55	4.39	81.02	79.55
RAW—Deformation (D1, D2) + Proximity	72.73	2.73	72.87	72.73
MAV—Deformation (D1, D2) + Proximity	80.29	3.84	81.22	80.29
RMS—Deformation (D1, D2) + Proximity	80.34	3.70	81.32	80.34

### 4.3. Real-Time Implementation

**Figure 12:**
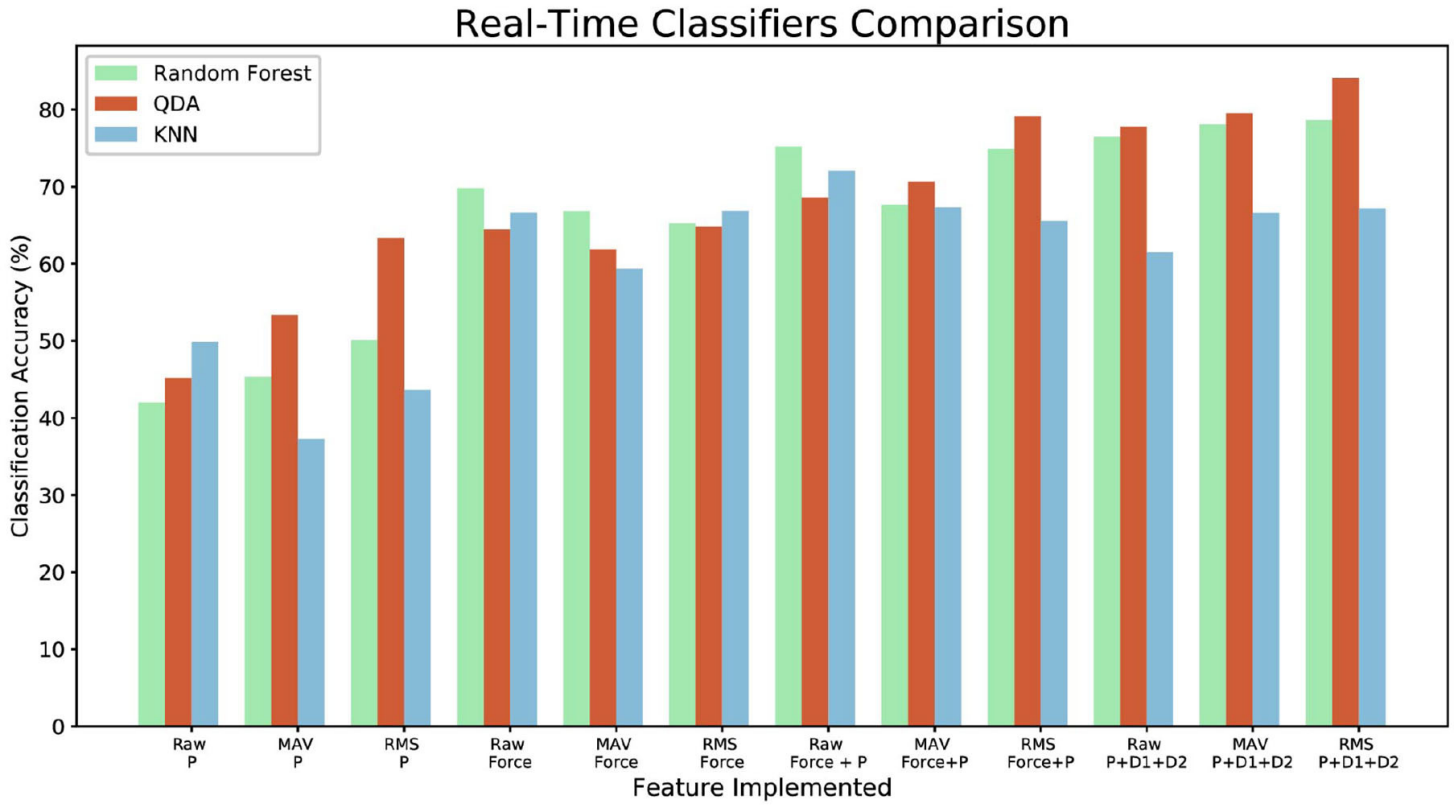
Real-time classification comparison for Random Forest, QDA, and KNN for the complete set of implemented features.

To further test the result of the classifier an online application was developed for the crack recognition analysis. During this experiment, it was found that the fiber optic cables position and their twisting influence the sensor data. Thus, an additional acquisition was necessary to obtain a model to use for the real-time classification. Offline models of Random Forest Classifier, KNN, and QDA were generated implementing the newly acquired data. The models were later used to predict the class of the data acquired in real-time while sliding the sensor over different surfaces. The software marks the start position of the detected crack and end position in relation to the Geomagic position. The same analysis, as the previously described offline classifier, was applied. Three continuous sliding movements were performed on each of the crack type surfaces as shown on the first row of Figure 2. Each movement was performed in a different section of the surface (top, center, bottom). The possible proximity data (only proximity, only deformation, deformation + proximity, and P + D1 + D2) and features (Raw Data, MAV, RMS) combination were investigated for each classifier, for a total of 432 classified movements. Figure 12 shows the results of the real time classification accuracy. In this case, the KNN classifier achieves better results than the Random Forest. Increasing the number of classified sliding movements may reduce this difference. Having 3D printing and of regular shape objects may limit the training and testing but this will be addressed and improved in future analysis.

## 5. Conclusion and Future Work

This work demonstrates how tactile and proximity sensing can be efficiently used to perform automatic crack detection. The proposed method uses machine learning techniques to detect the surface fractures and bumps of explored objects based on fiber optical proximity signals which are recorded during physical interaction between a custom-designed robotic finger and the remote environment. Experimental validation of the proposed method has shown that it is possible to achieve around 94% for crack detection and 80% for crack width classification accuracy. To achieve better results for the crack width classification, an alternative regressor may be more appropriate to use with respect to the implemented classifier. Real-time classification results, on three sliding movements, shows that it is possible to correctly characterize the surface of the investigated object. During this experiment, it was found that the fiber optic cables position and their twisting influences the sensor data. Thus, additional analysis will be required to compensate for the change of flow of the data. In contrast to previous techniques, which rely on visual modality, the proposed approach, based on optical fibers, which may be more suitable for operation in extreme environments (such as nuclear facilities) where radiation damages electronic components of video cameras.

Future research will focus on integrating a multi-modal approach with visual patches and implementation of the proposed system on a teleoperated mobile manipulation system. We plan to demonstrate how automatic fracture characterization will be efficiently integrated with the mobile manipulator controller (Farkhatdinov and Ryu, 2008) and how the obtained tactile data can be visualized in a dedicated virtual reality-based human-operator interface (Omarali et al., 2020). Further studies will be performed on dimensionality reduction with principal component analysis which may increase the classification accuracy. Additional features, such as local min-max values, which may give a better comprehension of the data, will be analyzed. The implementation of an alternative bio-inspired ciliary force sensor will be investigated for small crack detection (Ribeiro et al., 2017).

## Data Availability Statement

The datasets generated for this study can be found in the figshare repository “Automatic Fracture Database” available at https://figshare.com/s/14deb00d874400e34d67.

## Author Contributions

FP developed the classification algorithm, experimental methods, performed the experiments, and wrote the draft of the paper. IF developed the experimental methods, contributed to the data analysis, and provided the input to the paper. KA advised on the experimental methods and corrected the paper. JK and SP assisted with the paper writing. All authors contributed to the article and approved the submitted version.

## Conflict of Interest

JK was employed by the company Ocado Technology. The remaining authors declare that the research was conducted in the absence of any commercial or financial relationships that could be construed as a potential conflict of interest.

## Footnotes

^1^https://www.syntouchinc.com/en/sensor-technology/

^2^https://figshare.com/s/14deb00d874400e34d67
